# The Importance of Mentorship in Thoracic Surgery Residency

**DOI:** 10.3390/jcm14207391

**Published:** 2025-10-20

**Authors:** Paola Ciriaco

**Affiliations:** Department of Thoracic Surgery, IRCCS San Raffaele Scientific Institute, 20132 Milan, Italy; ciriaco.paola@hsr.it

**Keywords:** thoracic surgery residency, mentorship, residents’ satisfaction

## Abstract

**Background:** Effective mentorship is a critical component of thoracic surgery training, fostering the acquisition of advanced clinical skills, professional identity, and academic growth essential for the development of future specialists. This perspective article examines the role of mentorship in thoracic surgery training, with a particular focus on the value perceived by residents. **Methods**: The research was conducted on Pubmed, using the following targeted search queries: ‘mentorship and thoracic residency’ and ‘thoracic surgery residents’ satisfaction’. Only studies published in English between 2015 and 2025 were included. Studies lacking primary data on mentorship outcomes were excluded. **Results**: Out of 286 studies initially identified, 25 fulfilled the inclusion criteria. Five main thematic domains emerged: *Impact and Effectiveness of Mentorship* (12 studies), *Training Pathways and Workforce* (13), *Diversity, Gender, and Equity* (8), *Resident Well-Being and Outcomes* (4), and *Assessment Tools* (1). Overlaps among domains were frequent, underscoring the multifaceted role of mentorship in thoracic surgery training. **Conclusions**: Mentorship is a key determinant of success in thoracic surgical training, enhancing technical skills, clinical performance, and academic productivity while protecting resident well-being. It also promotes diversity, equity, and inclusion, supports workforce sustainability, and strengthens professional development. Evidence confirms that mentorship should be considered an essential component of training curricula, with future efforts directed toward structured, equitable, and measurable mentorship programs.

## 1. Introduction

Mentorship is widely recognized as a cornerstone of surgical education, contributing to the acquisition of technical competence, refinement of clinical judgment, and development of professional identity [1,2]. Across surgical disciplines, the value of mentorship is consistently emphasized. In thoracic and cardiothoracic surgery, where technical complexity and the demands of high-stakes decision-making are particularly pronounced, mentors play an especially pivotal role. They serve not only as teachers in the operating room but also as role models, advisors, and advocates, guiding trainees through procedural learning, scholarly development, and long-term career planning [3].

The scope of mentorship in thoracic surgery extends beyond technical instruction. From the outset of training, residents must navigate steep learning curves, master intricate operative techniques while simultaneously cultivating the capacity to make nuanced intraoperative and perioperative decisions. In this context, mentors provide structured feedback, foster safe skill acquisition, and model the professional composure required in high-pressure situations. Outside the operating theater, they are equally important in orienting trainees to academic surgery—facilitating research opportunities, offering guidance on manuscript preparation and grant writing, and fostering connections within national and international societies. This dual contribution, encompassing both technical and academic development, positions mentorship as a central determinant of a resident’s trajectory within the specialty.

The influence of mentorship is not confined to professional skill-building. Many residents report that their mentors shape broader aspects of their personal and professional lives, providing counsel on resilience, work–life integration, and the navigation of complex career decisions. Particularly in a specialty known for its demanding training environment, this psychosocial dimension of mentorship is highly valued. Effective mentors demonstrate empathy, accessibility, and advocacy, offering reassurance and modeling sustainable approaches to a surgical career. As such, mentorship fosters not only competence but also a sense of belonging and purpose within the field.

Despite these benefits, mentorship experiences in thoracic surgery remain heterogeneous. Although most residents report having at least one mentor, the quality and consistency of these relationships vary considerably [3,4]. In many programs, mentorship arises informally through shared clinical experiences, while others rely on structured initiatives with variable success. Commonly reported challenges include the lack of institutionally supported mentorship frameworks, limited formal training for faculty mentors, and suboptimal mentor–mentee matching processes. Relationships that lack alignment in interests or expectations may result in superficial or ineffective interactions, diminishing the potential of mentorship to positively influence a trainee’s development.

Contemporary changes in surgical education further complicate the mentorship landscape. Duty-hour restrictions, increasing case complexity, and heightened focus on patient safety have reduced the duration and intimacy of mentor–trainee interactions traditionally afforded by apprenticeship-style training. Simultaneously, emerging modalities—such as simulation-based education, robotic-assisted surgery, and tele-mentoring—have created new opportunities for teaching and mentorship that extend beyond the confines of the operating room. Residents increasingly express a desire for mentorship models that integrate the values of traditional apprenticeship with these contemporary approaches, emphasizing structured feedback, flexibility, and broader access to role models.

From the residents’ perspective, mentorship is defined not only by technical instruction but also by the quality of the interpersonal relationship. Trainees consistently highlight attributes such as approachability, investment in mentee success, and open communication as central to effective mentorship. They often seek role models whose professional paths mirror their aspirations, but also value mentors who actively foster inclusivity and support individual well-being. Importantly, the presence of effective mentorship has been linked to enhanced resident satisfaction, increased academic productivity, and a stronger commitment to careers in academic surgery. These findings underscore the central role mentorship plays in shaping both individual careers and the overall vitality of the specialty.

In recognition of these challenges, some training programs have implemented structured mentorship initiatives. These may include formal mentor–mentee pairing processes, faculty development workshops on effective mentorship strategies, and regular programmatic evaluations of mentorship effectiveness. Early evidence suggests that such initiatives can improve resident satisfaction and foster stronger academic engagement. However, their success depends on active participation, thoughtful pairing, and institutional cultures that value mentorship as an essential component of surgical training rather than an optional supplement. Moreover, mentorship should be viewed as dynamic, evolving in response to the resident’s stage of training—from technical guidance in early years to career sponsorship and leadership development as independence approaches.

This perspective article seeks to analyze the role of mentorship in thoracic surgery while foregrounding the resident viewpoint. By examining how trainees perceive mentorship, what qualities they value, and the barriers they face, we aim to clarify both the strengths and the gaps within current mentorship structures. Ultimately, strengthening mentorship in thoracic surgery represents an investment not only in individual residents but also in the specialty. Robust mentorship fosters technical excellence, nurtures scholarly productivity, supports personal well-being, and ensures the continued advancement of thoracic surgery as a dynamic, inclusive, and sustainable field.

## 2. Materials and Methods

A systematic literature search was conducted in PubMed to identify studies examining mentorship in thoracic surgery training. The search strategy employed a combination of targeted keywords and Boolean operators to ensure comprehensive coverage of the relevant literature. Search terms included“ *mentorship and thoracic residency*”, “*mentorship programs in thoracic surgery*” and “*thoracic surgery residents’ satisfaction*” among others, to capture studies addressing both formal and informal mentorship experiences. The search was restricted to studies published in English between 2015 and 2025, with full-text availability to facilitate detailed evaluation of study methodology, outcomes, and conclusions.

All study designs were considered eligible for inclusion, encompassing original research articles, cross-sectional and longitudinal surveys, qualitative investigations, and systematic or narrative reviews. This inclusive approach was intended to capture the full spectrum of evidence regarding mentorship structures, mentor–mentee relationships, and the professional experiences of thoracic surgery residents. Articles were initially screened by title and abstract for relevance, followed by full-text review for those meeting preliminary inclusion criteria. Studies were included if they examined structured mentorship programs, informal mentorship interactions, or residents’ perceptions, satisfaction, and professional development related to mentorship.

Exclusion criteria encompassed studies lacking primary data on mentorship outcomes or those unrelated to thoracic surgery training. Key data were extracted from eligible studies, including study design, sample characteristics, mentorship model, outcomes assessed, and major findings relevant to the influence of mentorship on resident training, career progression, and satisfaction.

Following the screening and data extraction process, the included studies were synthesized to provide a comprehensive overview of contemporary mentorship practices and their perceived impact on thoracic surgery trainees. A summary of the studies included in this review is presented in Table 1, outlining study characteristics, methodological approaches, and principal findings. This synthesis aims to inform future mentorship initiatives and highlight areas for improvement in fostering effective mentor–mentee relationships within thoracic surgery residency programs.

## 3. Results

**Figure 1:**
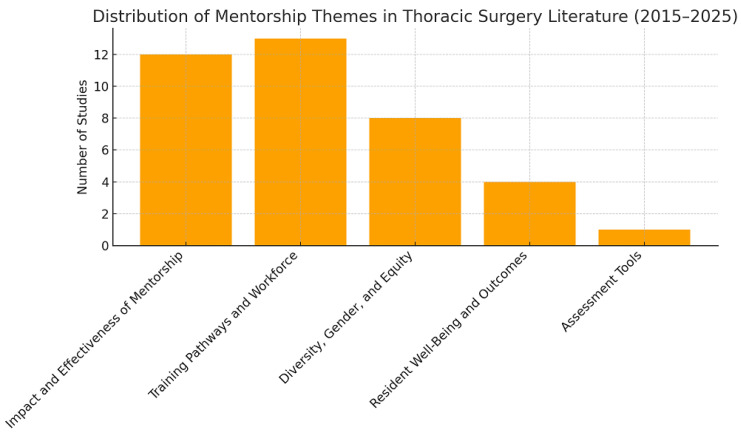
PubMed research results.

From an initial yield of 286 potentially relevant studies, 25 met the inclusion criteria and were incorporated into the final analysis. The literature review identified five principal domains most directly associated with mentorship in thoracic surgery training: “*Impact and Effectiveness of Mentorship*”, “*Diversity, Gender, and Equity*”, “*Training Pathways and Workforce*”, “*Resident Well-Being and Outcomes*”, and “*Assessment Tools*”. Importantly, these domains were not mutually exclusive; rather, they frequently overlapped, reflecting the multifaceted nature of mentorship in surgical education. Among the included studies, “*Impact and Effectiveness of Mentorship*” emerged most prominently, addressed in 12 studies. “*Training Pathways and Workforce*” was discussed in 13 papers, while “*Diversity, Gender, and Equity*” was the central theme in eight. “*Resident Well-Being and Outcomes*” appeared as the primary focus in four articles, and one study specifically examined “*Assessment Tools*” (Figure 1).

The studies under the *Impact and Effectiveness of Mentorship* domain consistently emphasized mentorship as a cornerstone of cardiothoracic surgical training. They highlighted mentorship as not only a vehicle for technical skills acquisition but also a driver of clinical judgment, professional identity formation, and long-term career development. Several studies argued that structured mentorship programs yield superior outcomes compared with ad hoc or informal arrangements, particularly in promoting academic productivity and research engagement. Collectively, these papers reinforced the view that mentorship exerts a dual influence: advancing resident performance and safeguarding their well-being.

The domain of *Training Pathways and Workforce* further illustrated mentorship’s pivotal role in shaping thoracic surgeons’ educational and professional journeys. Mentorship was shown to extend beyond the transmission of operative expertise, encompassing guidance in decision-making, research, and career planning. Evidence from these studies demonstrated tangible benefits, including enhanced clinical competence, increased scholarly output, and improved readiness for independent practice. Importantly, mentorship was also framed as an institutional imperative, influencing recruitment and retention in an era where maintaining a robust cardiothoracic workforce remains challenging.

Papers categorized under *Diversity, Gender, and Equity* underscored the transformative potential of mentorship in fostering inclusivity within surgery. These studies noted that targeted mentorship initiatives, such as same-gender or culturally concordant pairings, could mitigate barriers historically faced by women and underrepresented minorities in thoracic surgery. However, they also highlighted persistent inequities, including disparities in sponsorship, recognition, and career advancement. This body of work emphasized that mentorship should not be viewed solely as an individual relationship but as a structural intervention capable of addressing systemic inequalities.

The theme of *Resident Well-Being and Outcomes* focused on the dual relationship between mentorship, trainee wellness, and clinical results. These studies illustrated that effective mentorship provides a buffer against burnout, supports resilience, and contributes to psychological safety—factors that directly impact both resident satisfaction and patient care. The literature reinforced the notion that mentorship should be conceptualized as a continuum, extending beyond technical training to encompass emotional and professional support throughout a surgeon’s career trajectory.

**Figure 2:**
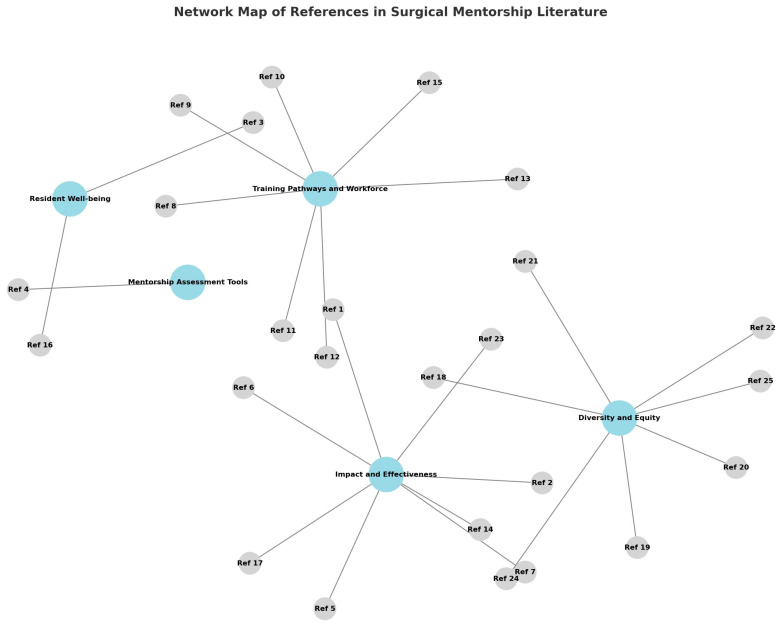
References clustered into five themes: “Impact and Effectiveness of Mentorship”, “Diversity, Gender, and Equity”, “Training Pathways and Workforce”, “Resident Well-Being and Outcomes”, and “Assessment Tools.

Finally, one study within the *Assessment Tools* domain introduced an innovative approach to evaluating mentorship by developing a “mentoring quiz” designed for surgical residents. This tool facilitated self-reflection on the quality of mentoring relationships and provided a framework for constructive dialogue between mentors and mentees. By moving beyond descriptive accounts of mentorship to measurable instruments, this study highlighted the potential for standardized assessment to guide program development and ensure accountability (Figure 2).

Taken together, the thematic analysis demonstrates that mentorship in thoracic surgery training is a multidimensional construction with wide-ranging implications. Its influence spans technical education, workforce sustainability, equity in representation, resident wellness, and even the culture of academic evaluation.

## 4. Discussion

### 4.1. Impact and Effectiveness of Mentorship

Mentorship is increasingly recognized as a fundamental element in the training of thoracic surgeons. Beyond the transmission of technical expertise, mentors provide guidance in clinical decision-making, research, and professional development [5,6]. A structured mentorship relationship fosters not only the acquisition of surgical skills but also the cultivation of critical thinking, resilience, and ethical standards. Mentors serve as role models, offering support during challenging clinical scenarios, facilitating reflection on complex cases, and promoting the development of professional judgment. For these reasons, mentorship represents a cornerstone in the formation of competent and well-rounded thoracic surgery specialists [7].

Evidence strongly supports mentorship as an essential component of surgical education, with particularly compelling data emerging from cardiothoracic training [8]. While study contexts, designs, and methodologies vary, their collective findings converge on a central theme: mentorship is not a peripheral element of training but a determinant of both individual and institutional success [9,10]. Reviews in general surgery and cardiothoracic surgery [1,2,11,12,13] consistently report that trainees with active mentors demonstrate superior clinical competence, greater academic productivity, and increased access to leadership opportunities. These benefits are most pronounced when mentorship is delivered through structured programs, characterized by defined goals, regular meetings, and measurable outcomes, as opposed to informal or ad hoc arrangements [5,13,14].

Structured mentorship also promotes sustained scholarly productivity. Trainees with mentors are more likely to engage in research, publish manuscripts, present at national meetings, and secure competitive fellowships, thereby expanding professional networks and laying the groundwork for future leadership within academic surgery. Additionally, structured mentorship contributes to career satisfaction, retention in the field, and the development of professional identity. In contrast, reliance on unstructured mentorship can result in variability in training quality, inequitable access to academic opportunities, and missed potential for career advancement [15,16]. Collectively, these findings underscore the critical role of mentorship as an integral component of thoracic surgery education, shaping not only technical proficiency but also long-term professional growth and success.

### 4.2. Diversity, Gender, and Equity

Mentorship has the potential to fundamentally reshape the culture of surgery with respect to diversity, equity, and inclusion (DEI). Beyond its traditional role in fostering technical growth and professional advancement, mentorship serves as a critical tool for addressing structural inequities that influence who enters, thrives, and leads in surgical careers. Multiple studies identify mentorship as a powerful mechanism to mitigate disparities in representation and career development. For example, same-gender mentoring has been shown to increase women’s sense of belonging, self-efficacy, and sustained engagement in surgical pathways—factors that directly influence recruitment and retention [17]. More broadly, mentorship frameworks that include underrepresented minorities highlight its role in providing tailored guidance, active sponsorship, and access to professional networks that are often restricted or exclusionary [18]. Early and meaningful exposure to mentors who reflect diverse identities not only helps normalize inclusion within the field but also creates pathways for skill development, research opportunities, and leadership preparation—helping to bridge gaps that have historically contributed to underrepresentation in surgical specialties.

Yet, despite these promising advances, inequities in opportunity and recognition persist. Patton et al. [19] demonstrated that women continue to receive significantly less sponsorship compared with men in NIH-funded contexts—a disparity that directly constrains opportunities for academic advancement, grant acquisition, and entry into leadership roles. Similarly, surveys conducted by Stephens et al. [13,20] underscore the persistence of gender-based inequities in satisfaction, promotion, and long-term career trajectories, reflecting systemic barriers that cannot be fully resolved through mentorship alone. These findings highlight a critical tension: while mentorship offers individual and collective benefits, its impact is limited when conceptualized solely as a dyadic, mentor–mentee exchange. To achieve meaningful change, mentorship must instead be understood as an institutional and cultural intervention with the capacity to reshape norms, structures, and expectations across the surgical community.

Targeted mentorship programs represent one avenue for this transformation. When intentionally designed to address systemic barriers, such programs can foster equitable access to advancement opportunities while amplifying the voices of individuals who remain marginalized within surgery. Formal structures, such as defined mentorship curricula, transparent selection processes, and accountability metrics, strengthen program effectiveness and guard against the perpetuation of implicit bias. Embedding diversity-focused objectives—such as ensuring representation among mentors, promoting cross-cultural competency, and incentivizing inclusive leadership—further amplifies impact. Collectively, these measures extend mentorship from an interpersonal tool to a lever of institutional change.

Integrating DEI-focused mentorship into the policy and culture of surgical departments is therefore essential for progress. By embedding mentorship into the formal scaffolding of training and career development, surgical institutions can advance equity, improve retention, and cultivate leaders who embody and champion inclusivity. Ultimately, such efforts not only create more equitable professional environments but also build a surgical workforce that more closely reflects—and is prepared to meet—the needs of the diverse populations it serves.

### 4.3. Training Pathways and Workforce

Mentorship plays a critical role in guiding trainees through their educational and professional development. Deivasigamani et al. [5] demonstrated that structured mentorship significantly improved success in the residency match, emphasizing the importance of early, organized mentor–mentee relationships. Similarly, studies among medical students [10,21] indicate that exposure to mentors early in training enhances understanding of cardiothoracic pathways, clarifies expectations for clinical and research competencies, and fosters sustained interest in the specialty. Programs such as the Thoracic Surgery Directors Association (TSDA) mentorship initiatives and institution-specific early exposure programs have shown that pairing students with faculty mentors increases engagement, supports informed decision-making, and encourages consideration of underrepresented groups in cardiothoracic surgery. Conversely, as noted by Koomalsingh and Mokadam [22], the absence of active mentorship can deter promising candidates, contribute to uncertainty regarding career paths, and potentially lead to declining interest in specific subspecialties, creating workforce gaps that may affect the field’s future.

For early-career surgeons, mentorship functions as a catalyst for securing academic positions, obtaining research funding, and developing leadership portfolios. Structured mentorship programs, such as formal faculty–resident pairings with clear goals and regular meetings, have been associated with increased research productivity, higher publication rates, and greater success in securing competitive fellowships. Mentors provide guidance on manuscript preparation, grant writing, networking, and professional visibility, all of which influence career trajectory. Effective mentorship ensures that trainees acquire not only technical proficiency but also the professional skills required for independent practice, leadership roles, and ethical decision-making. Programs that integrate multidisciplinary mentorship, including research and clinical faculty, have been particularly successful in fostering holistic professional development and preparing trainees for academic and administrative responsibilities.

In contrast, inadequate mentorship may limit career advancement, reduce academic output, and hinder opportunities for leadership. Trainees without strong mentorship may experience slower progression, fewer publications, and limited access to professional networks, which can impact retention within the specialty. Collectively, these findings underscore that mentorship is a foundational component of cardiothoracic training, shaping both individual success and the broader development of the specialty. Institutions that prioritize structured, proactive mentorship programs are better positioned to cultivate highly skilled, motivated, and well-rounded surgeons capable of advancing clinical care, research, and leadership in cardiothoracic surgery.

### 4.4. Resident Well-Being and Outcomes

Equally noteworthy is the impact of mentorship on resident well-being. Burnout has been repeatedly identified as a critical threat to surgical training, with significant implications for both patient safety and workforce sustainability. Gaeta et al. and Mattioni et al. [3,15] provide complementary perspectives, demonstrating that mentorship functions as a protective factor against burnout, emotional exhaustion, and professional disengagement, whereas its absence correlates with higher levels of stress, dissatisfaction, and attrition among surgical trainees. By fostering supportive mentor–mentee relationships, residents gain access to guidance, encouragement, and coping strategies that help them navigate the demands of rigorous training. These findings, together with Sbrocchi and Denlinger’s [16] assertion that “mentoring never ends”, emphasize that mentorship should be conceptualized as a continuous process that extends beyond residency, offering long-term support for career resilience, professional fulfillment, and work–life integration.

Structured mentorship programs targeting resident wellness have been implemented in several surgical institutions. For example, some programs incorporate formal wellness check-ins, peer mentoring, and faculty mentorship focused on stress management, time prioritization, and career guidance. These initiatives provide protected time for mentorship discussions, normalize conversations around mental health, and actively promote resilience. Residents supported by such programs are more likely to acknowledge mistakes, seek constructive feedback, and engage in reflective learning, thereby fostering psychological safety. This environment encourages personal growth, reinforces accountability, and improves patient care. Furthermore, mentorship-driven wellness initiatives contribute to stronger team cohesion, effective communication, and a positive institutional culture, creating a sustainable foundation for both trainee well-being and the long-term success of surgical programs.

### 4.5. Assessment Tools

Finally, mentorship significantly impacts academic culture and can be systematically measured through structured assessment tools. As demonstrated by Stephens [13], effective mentorship promotes higher scholarly output, broader professional networks, and deeper integration into the academic community, thereby reinforcing the educational and research missions of surgical departments. Conversely, disparities in conference participation and authorship, as documented by Ries [23], illustrate that without equitable mentorship and sponsorship, structural imbalances in visibility, recognition, and career advancement persist, particularly for underrepresented trainees. To address these challenges, structured tools such as mentoring quizzes, evaluation forms, and longitudinal feedback assessments have been developed. These instruments encourage both mentors and mentees to engage in self-reflection, identify areas for growth, and monitor the effectiveness of mentorship over time [24]. By providing measurable insights, these tools facilitate continuous improvement of mentorship programs, ensuring they remain equitable, productive, and aligned with the goals of fostering academic success and professional development across all trainees.

## 5. Conclusions

Mentorship has repeatedly been identified as one of the most powerful determinants of success in surgical training. Far from being a peripheral relationship, it is increasingly regarded as a structural element of surgical education. Evidence from multiple studies demonstrates that effective mentorship enhances technical skill acquisition, accelerates the learning curve, and improves overall clinical performance. In both cardiothoracic and general surgery, trainees who report active and engaged mentors consistently achieve higher levels of competence, demonstrate greater autonomy in the operating room, and transition more confidently into independent practice. In parallel, mentorship fosters academic productivity by facilitating research involvement, guiding manuscript preparation, and providing access to scholarly networks.

The influence of mentorship, however, extends well beyond technical and academic outcomes. Resident well-being—an area of growing concern in surgical education—is significantly strengthened by the presence of effective mentors. By providing emotional support, perspective, and advocacy, mentorship has been shown to mitigate burnout, enhance resilience, and promote professional identity formation during the most demanding phases of training. These benefits often persist beyond residency, supporting surgeons as they assume leadership roles and navigate the early years of independent practice. In this sense, mentorship functions as a continuum, shaping not only the trajectory of training but also the durability and satisfaction of an entire career.

Equally important is the role of mentorship as a driver of diversity, equity, and inclusion within surgery. Systemic barriers have historically limited the representation of women and underrepresented minorities in cardiothoracic surgery. Targeted mentorship initiatives provide a means of countering these inequities by fostering belonging, offering role models, and creating opportunities for advancement that might otherwise remain inaccessible. Same-gender and culturally concordant mentorship programs have been shown to increase recruitment, retention, and career satisfaction among underrepresented groups. As such, mentorship is not merely supportive on an individual level but transformative at an institutional and cultural scale, promoting inclusivity across the surgical workforce.

Recruitment and workforce sustainability represent another domain in which the impact of mentorship is clearly demonstrated. Medical students and junior residents who are exposed to effective mentors are more likely to consider surgical careers, succeed in competitive application processes, and remain engaged within the specialty. Conversely, the absence of mentorship has been identified as a risk factor for attrition, dissatisfaction, and decreased specialty attractiveness. In an era where recruitment into demanding subspecialties such as cardiothoracic surgery remains a challenge, mentorship functions as a strategic mechanism for sustaining interest and cultivating the next generation of surgeons.

Finally, mentorship plays a formative role in shaping academic culture. It not only drives scholarly output but also builds durable professional networks and creates pathways for sponsorship. Mentors often serve as advocates who nominate trainees for presentations, awards, and leadership opportunities, thereby addressing inequities in visibility and recognition. In doing so, mentorship strengthens the academic mission of surgical departments while ensuring that advancement is distributed more equitably.

Taken together, the evidence confirms that mentorship is not an optional adjunct to surgical education but a structural necessity. Its impact spans technical training, resident well-being, diversity and inclusion, recruitment, and academic advancement. Future efforts must therefore focus on institutionalizing mentorship within surgical curricula. This includes creating formalized programs, training faculty in effective mentoring practices, and embedding mechanisms for accountability and outcome measurement. By ensuring equitable access to high-quality mentorship and evaluating its effects through measurable frameworks, surgical education can optimize trainee development while simultaneously securing the sustainability of the specialty. Ultimately, a deliberate commitment to mentorship will cultivate a workforce that is not only technically competent and academically productive but also resilient, inclusive, and prepared to lead the evolving field of surgery.

## Figures and Tables

**Table 1:** Inclusion and Exclusion Criteria.

Database	PubMed
**Search queries**	“Mentorship and thoracic residency”; “thoracic surgery residents’ satisfaction”
**Language**	English
**Publication type** **Time interval:**	Original research, surveys, reviews 2015–2025
**Inclusion criteria**	Studies focusing on mentorship programs, mentor–mentee relationships, or residents’ perceptions of mentorship in thoracic surgery
**Exclusion criteria**	Studies lacking primary data on mentorship outcomes
**Screening process**	Titles and abstracts screened for relevance; full texts reviewed when available
**Outcome**	Selected studies analyzed to summarize the role and perceived importance of mentorship in thoracic surgery training

## Data Availability

No new data were created.
